# Combining Hepatic Percutaneous Perfusion with Ipilimumab plus Nivolumab in advanced uveal melanoma (CHOPIN): study protocol for a phase Ib/randomized phase II trial

**DOI:** 10.1186/s13063-022-06036-y

**Published:** 2022-02-13

**Authors:** T. M. L. Tong, M. K. van der Kooij, F. M. Speetjens, A. R. van Erkel, R. W. van der Meer, J. Lutjeboer, E. L. van Persijn van Meerten, C. H. Martini, R. W. M. Zoethout, F. G. J. Tijl, C. U. Blank, M. C. Burgmans, E. Kapiteijn

**Affiliations:** 1 Department of Medical Oncology, Leiden University Medical Center, PO Box 9600, 2300 RC Leiden, The Netherlands; 2 Department of Radiology, Leiden University Medical Center, PO Box 9600, 2300 RC Leiden, The Netherlands; 3 Department of Anesthesiology, Leiden University Medical Center, PO Box 9600, 2300 RC Leiden, The Netherlands; 4 Department of Extra Corporal Circulation, Leiden University Medical Center, PO Box 9600, 2300 RC Leiden, The Netherlands; 5 Department of Medical Oncology, The Netherlands Cancer Institute – Antoni van Leeuwenhoek Hospital (NKI-AVL), PO Box 90203, 1006 BE Amsterdam, The Netherlands

**Keywords:** Advanced uveal melanoma, Percutaneous hepatic perfusion, Immunotherapy, Liver metastases

## Abstract

**Background:**

While immune checkpoint inhibition (ICI) has revolutionized the treatment of metastatic cutaneous melanoma, no standard treatments are available for patients with metastatic uveal melanoma (UM). Several locoregional therapies are effective in the treatment of liver metastases, such as percutaneous hepatic perfusion with melphalan (M-PHP). The available literature suggests that treatment with ICI following locoregional treatment of liver UM metastases can result in clinical response. We hypothesize that combining M-PHP with ICI will lead to enhanced antigen presentation and increased immunomodulatory effect, improving control of both hepatic and extrahepatic disease.

**Methods:**

Open-label, single-center, phase Ib/randomized phase II trial, evaluating the safety and efficacy of the combination of M-PHP with ipilimumab (anti-CTLA-4 antibody) and nivolumab (anti-PD-1 antibody) in patients with unresectable hepatic metastases of UM in first-line treatment, with or without the limited extrahepatic disease. The primary objective is to determine the safety, toxicity, and efficacy of the combination regimen, defined by maximum tolerated dose (MTD) and progression-free survival (PFS) at 1 year. Secondary objectives include overall survival (OS) and overall response rate (ORR). A maximum of 88 patients will be treated in phase I and phase II combined. Baseline characteristics will be described with descriptive statistics (*t*-test, chi-square test). To study the association between risk factors and toxicity, a logistic regression model will be applied. PFS and OS will be summarized using Kaplan-Meier curves.

**Discussion:**

This is the first trial to evaluate this treatment combination by establishing the maximum tolerated dose and evaluating the efficacy of the combination treatment. M-PHP has shown to be a safe and effective treatment for UM patients with liver metastases and became the standard treatment option in our center. The combination of ICI with M-PHP is investigated in the currently described trial which might lead to a better treatment response both in and outside the liver.

**Trial Registration:**

This trial was registered in the US National Library of Medicine with identifier NCT04283890. Registered as per February 2020 - Retrospectively registered.

EudraCT registration number: 2018-004248-49.

Local MREC registration number: NL60508.058.19.

## Administrative information

Note: the numbers in curly brackets in this protocol refer to SPIRIT checklist item numbers. The order of the items has been modified to group similar items (see http://www.equator-network.org/reporting-guidelines/spirit-2013-statement-defining-standard-protocol-items-for-clinical-trials/).

**Table:** 

Title {1}	Combining Hepatic Percutaneous Perfusion with Ipilimumab plus Nivolumab in advanced Uveal Melanoma (CHOPIN): Study protocol for a phase Ib/randomized phase II Trial.
Trial registration {2a and 2b}.	U.S. National Library of Medicine: NCT04283890 EudraCT registration number: 2018-004248-49
Protocol version {3}	Version 06, December 2020
Funding {4}	In kind contribution from Bristol-Myerss Squibb and Delcath Systems Inc.
Author details {5a}	T.M.L. Tong^1,2^, M.K. van der Kooij^1^, F.M. Speetjens^1^, A.R. van Erkel^2^, R.W. van der Meer^2^, J. Lutjeboer^2^, E.L. van Persijn van Meerten^2^, C.H. Martini^3^, R.W.M. Zoethout^3^, F.G.J. Tijl^4^, C.U. Blank^1,5^, M.C. Burgmans^2^, E. Kapiteijn^1^ 1) Department of Medical Oncology, Leiden University Medical Center, PO Box 9600, 2300 RC Leiden, The Netherlands 2) Department of Radiology, Leiden University Medical Center, PO Box 9600, 2300 RC Leiden, The Netherlands 3) Department of Anesthesiology, Leiden University Medical Center, PO Box 9600, 2300 RC Leiden, The Netherlands 4) Department of Extra Corporal Circulation, Leiden University Medical Center, PO Box 9600, 2300 RC Leiden, The Netherlands 5) Department of Medical Oncology, The Netherlands Cancer Institute – Antoni van Leeuwenhoek Hospital (NKI-AVL), PO Box 90203, 1006 BE Amsterdam, The Netherlands Corresponding author: Ms. T.M.L. Tong, T.M.L.Tong@lumc.nl
Name and contact information for the trial sponsor {5b}	Leiden University Medical Center Prof. Dr. A.J. Gelderblom Head of the department of Medical Oncology, C-7-P PO Box 9600, 2300 RC Leiden Email: A.J.Gelderblom@lumc.nl
Role of sponsor {5c}	This is an investigator-initiated trial. Leiden University Medical Center is the only participating center. Bristol-Myers Squibb contributes to the study with the supply of ipilimumab and nivolumab. Delcath Systems Inc. contributes to the study by supplying the kits for the percutaneous hepatic perfusion procedure.

## Introduction

### Background and rationale {6a}

Uveal melanoma (UM) is the most common intraocular malignant tumor in adults [1]. It is a rare type of malignancy, with an incidence of 4–7 cases per million in Europe [2]. Treatment options for the primary tumor include radiotherapy and enucleation [3], but even after successful treatment of the primary tumor approximately half of all patients will develop metastases in the following years [3, 4]. UM has a remarkable dissemination pattern, spreading purely hematogenously, with the liver as dominant site. Approximately 90% of patients with metastatic disease have liver involvement and in the majority of patients, this is initially the only organ with detectable disease [5]. Prognosis of metastatic UM remains dismal and has improved little over the last 30 to 40 years [1], since no standard treatment is available. Therefore, the need for better treatment remains.

Immune checkpoint inhibitors (ICI) improved overall survival (OS) in metastatic cutaneous melanoma (CM) in phase III studies [6–8], but seem to have limited effect as monotherapy in metastatic UM [9–11]. UM and CM are biologically two distinct tumor types, both having a different set of driver mutations. Furthermore, UM has a lower mutational load as compared to CM, leading to limited neoantigen presentation. Additionally, there is lower PD-1 and PD-L1 expression in patients with UM compared to CM [12]. The aforementioned differences are most probably the reasons that systemic treatment in UM is not as successful as in CM. A retrospective analysis identified 2 out of 12 UM patients that achieved a partial response (PR) after being treated with ipilimumab (anti-CTLA4 monoclonal antibody) and nivolumab (anti-PD-1 monoclonal antibody) [13]. Interestingly, both patients in this study received a form of liver-directed therapy (selective internal radiation therapy; SIRT) and chemoembolization) prior to ICI therapy. Recent studies investigating combined ICI in metastatic UM patients have reported promising results, with overall response rates (ORR) varying from 11.5 to 18% [14–16].

Considering the metastatic pattern, locoregional liver treatments play an important role. Several studies have indeed shown the benefit of locoregional treatment in patients with liver-dominant UM metastases. Locoregional treatment options range from surgical resection and ablation to transarterial therapies, such as immunoembolisation, radioembolization, chemoembolization, and hepatic perfusion [17]. A promising novel liver-directed therapy is percutaneous hepatic perfusion with melphalan (M-PHP). A randomized phase III trial for patients with liver-dominant CM and UM metastases demonstrated the superiority of M-PHP over best alternative care [18]. Additionally, a recent non-randomized phase II trial showed marked response of hepatic metastatic lesions in the majority of patients. Despite the good response, 74% of patients developed extrahepatic disease during follow-up, whereas the liver metastases were mainly stable or had regressed [19].

M-PHP causes cell necrosis through deoxyribonucleic acid (DNA) damage, which may evoke immunomodulation and enhance antigen presentation. ICIs also induce an immunomodulatory effect via a different mechanism. Anti-CTLA-4 antibodies boost T cell activation and the following anti-tumor response by blocking the interaction of CTLA-4 and CD80/86 and inducing immune responses to weak tumor antigens. Antibodies against PD-1 or its ligand PD-L1 prevent the inactivation of tumor-reactive immune cells [2, 12]. Considering these mechanisms we hypothesize that the combination of M-PHP and ICI could lead to improved hepatic and extrahepatic disease control through a synergistic effect.

In this trial, the combination therapy of M-PHP with ICI is investigated with the use of immunotherapeutic agents ipilimumab and nivolumab. The maximum tolerated dose (MTD) of ipilimumab and nivolumab in combination with M-PHP will be established in a phase Ib study. The following randomized phase II study will determine the efficacy of combination treatment of M-PHP with ipilimumab and nivolumab compared to M-PHP alone.

### Objectives {7}

The primary objective of phase Ib is to determine the safety and toxicity of the combination of M-PHP with ipilimumab and nivolumab. Based on the number of dose limiting toxicities (DLTs), the MTD and recommended phase II dose of the combination treatment will be determined. In the randomized phase II part, the primary objective is to evaluate the efficacy of combination treatment of M-PHP with ipilimumab plus nivolumab.

Secondary objectives are to determine OS, best ORR, overall clinical response according to Response Evaluation Criteria in Solid Tumors version 1.1 (RECIST 1.1) and Immune Response Evaluation Criteria In Solid Tumors (irRECIST), and duration of response for patients achieving an objective response.

### Trial design {8}

This is an open-label, single-center, phase Ib/randomized controlled phase II trial, evaluating the safety and efficacy of the combination of M-PHP with ipilimumab and nivolumab in patients with metastasized uveal melanoma in the Netherlands. The superiority of the combination of M-PHP with ipilimumab and nivolumab will be compared to M-PHP only, assuming superiority of the combination therapy.

## Methods: Participants, interventions, and outcomes

### Study setting {9}

The study will be conducted according to the principles of the Declaration of Helsinki (Declaration of Helsinki, 64^th^ WMA General Assembly, Fortaleza, Brazil, October 2013) and in accordance with the Dutch Medical Research Involving Human Subjects Act (WMO). The protocol has been written, and the study will be conducted according to the ICH Harmonized Tripartite Guideline for Good Clinical Practice (GCP). The study protocol was approved by the Central Committee on Research Involving Human Subjects (CCMO), the Competent Authority (CA) and the Medical Research Ethics Committee (MREC) of Leiden, The Hague, and Delft and will be performed at the Leiden University Medical Center (LUMC) in the Netherlands.

### Eligibility criteria {10}

**Fig. 1:**
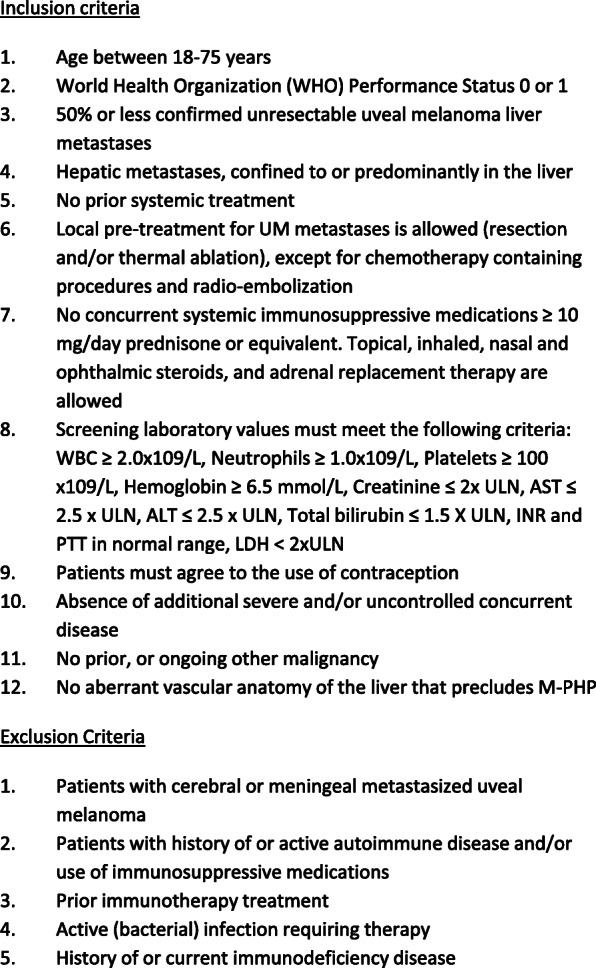
Inclusion and exclusion criteria. WHO, World Health Organization; UM, uveal melanoma; WBC, white blood cell; AST, aspartate aminotransferase; ULN, upper limit of normal; ALT, alanine aminotransferase; INR, international normalized ratio; PTT, partial thromboplastin time; LDH, lactate dehydrogenase; M-PHP, percutaneous hepatic perfusion with melphalan

Patients with unresectable hepatic metastases of UM, with or without limited extrahepatic disease. The main study inclusion and exclusion criteria are depicted in Fig. 1.

### Who will take informed consent? {26a}

The written patient information and consent form is given to each patient by his or her treating physician. It is the responsibility of the investigator to obtain signed informed consent (IC) from every patient prior to the registration in the study and the start of any study-related procedure. This will be done in accordance with the national and local regulatory requirements. The IC procedure will conform to the ICH guidelines on Good Clinical Practice. This implies that “the written IC form will be signed and personally dated by the patient or by the patient’s legally acceptable representative.”

### Additional consent provisions for collection and use of participant data and biological specimens {26b}

Patients are informed in the IC form of the additional blood drawings and biopsies that will be performed for the study. In the IC form, there is also mentioned that data is stored for 15 years for additional studies if needed. Patients have the option to agree or not agree to this, this will not have any effect on their participation in the CHOPIN trial.

## Interventions

### Explanation for the choice of comparators {6b}

In this trial, we will assess the effect of combined treatment with ipilimumab and nivolumab in UM patients. This treatment combination has been approved as standard therapy for irresectable late-stage CM in the Netherlands, based on superior response rate and progression-free survival from a randomized phase III trial [7]. While a lower rate of response to ipilimumab/nivolumab treatment has been described in UM patients [13], more recent trials [14–16] show promising results. The other intervention assessed in the CHOPIN trial is the M-PHP procedure. This is a novel intervention, but the superiority of M-PHP over standard available therapy has been demonstrated in a randomized controlled multicenter phase III trial for patients with liver metastases from CM and UM [18]. As UM is often associated with isolated diffuse (and thus unresectable) hepatic disease and effective systemic therapies are limited, M-PHP has been increasingly performed in these patients over the last two decades [18–23]. Combining M-PHP with checkpoint inhibitors could together lead to control of the hepatic and extrahepatic disease. In the randomized phase, the efficacy of the combination of M-PHP with ICI will be compared to M-PHP only.

### Intervention description {11a}

**Fig. 2:**
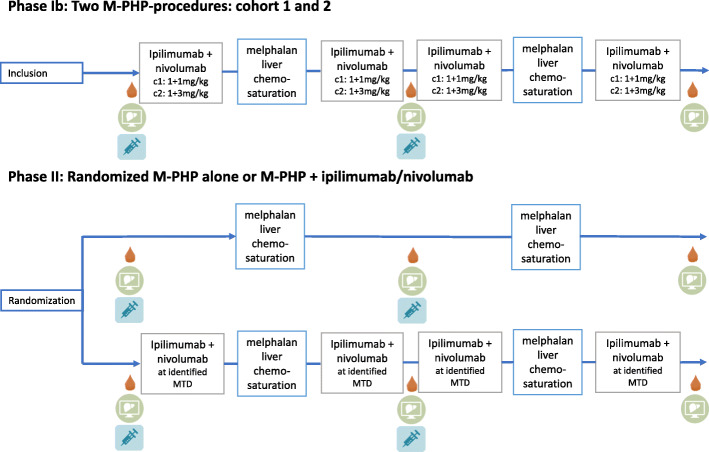
Treatment scheme. C1, Cohort 1; C2, Cohort 2; CT th/abd, CT-scan of the chest and abdomen; MRI, magnetic resonance imaging; Chemosaturation, percutaneous hepatic perfusion; , PBMC; , CT Th/abd, MRI liver; , tumor biopsy

The phase Ib part of the study will evaluate the safety and determine the MTD of the combination of M-PHP with ipilimumab and nivolumab in a 3+3 design, consisting of two dose cohorts of each three patients (Fig. 2). The first cohort in the phase Ib part will start with ipilimumab 1 mg/kg and nivolumab 1 mg/kg followed by M-PHP (3 mg/kg melphalan with a maximum dose of 220 mg). Subsequently, two courses of ipilimumab 1 mg/kg and nivolumab 1 mg/kg will be administered, followed by the second M-PHP and a last course of ipilimumab 1 mg/kg and nivolumab 1 mg/kg. In case of bone marrow toxicity ≥ grade 3 (according to the Common Terminology Criteria for Adverse Events version 4.03 (CTCAE v 4.03)) after the first M-PHP-procedure, a dose reduction of 25% will be applied at the second M-PHP. Treatment of the last patient in the first cohort and the first patient in the second should be at least 12 weeks apart.

In the second cohort of the phase Ib part the ICI dose will be escalated from 1 mg/kg both medications to ipilimumab 1 mg/kg and nivolumab 3 mg/kg. Besides that, the treatment scheme will be similar to the first cohort (Fig. 2). After determining the MTD, the randomized phase II part of the study will be opened. Randomization on a 1:1 basis will take place between M-PHP with ipilimumab and nivolumab versus M-PHP alone. In the phase II part, the sequence of treatments for the M-PHP with ipilimumab and nivolumab arm will be identical to the phase Ib part (Fig. 2), as well as the melphalan dose.

### Criteria for discontinuing or modifying allocated interventions {11b}

Treatment should be permanently discontinued in case of any drug/device-related liver function test (LFT) abnormality that meets the following criteria: AST or ALT >8× upper limit of normal (ULN), total bilirubin >5× ULN, concurrent AST or ALT >3× ULN and total bilirubin >2× ULN. Any grade 4 drug/device-related adverse event (AE) or laboratory abnormality requires discontinuation, except for grade 4 bone marrow toxicity which has restored to grade ≤1 or baseline value at the time of the second M-PHP procedure. In the case of grade 3 or 4 bone marrow toxicity (CTCAE v 4.03) after the first M-PHP procedure, a melphalan dose reduction of 25% will be performed at the second M-PHP. Additionally, any adverse event, laboratory abnormality, or intercurrent illness which, in the judgment of the investigator, presents a substantial clinical risk to the subject with continued nivolumab or ipilimumab dosing and/or melphalan/PHP will be a reason for discontinuation of the trial. If grade 3 or 4 immune-related AE (irAE) occurs, immunotherapy will be discontinued but patients will still be eligible for treatment with M-PHP according to the study scheme. In case of unexpected toxicity, the sponsor will discuss premature termination of the study with the subsidizing parties Bristol-Myers Squibb (BMS) and Delcath Systems Inc as well as the MREC.

### Strategies to improve adherence to interventions {11c}

All patients are adequately informed of the aims of the study, possible side effects, procedures, and possible hazards to which he/she will be exposed, as well as the mechanism of treatment allocation.

### Relevant concomitant care permitted or prohibited during the trial {11d}

As previously stated in the in- and exclusion criteria patients with concurrent medical conditions requiring the use of immunosuppressive medications, or immunosuppressive doses of systemic corticosteroids ≥ 10mg/day prednisone or equivalent, are not eligible. Use of systemic corticosteroids at <10 mg daily prednisone equivalent is allowed. Additionally, topical, inhaled, nasal and ophthalmic steroids, and adrenal replacement therapy are also allowed. Furthermore, it is not allowed to use other investigational drugs before study drug administration for systemic malignancy. Pregnant or nursing patients cannot participate.

### Provisions for post-trial care {30}

The sponsor has an insurance that is in accordance with the legal requirements in the Netherlands (Article 7 WMO). This insurance provides cover for damage to research subjects through injury or death caused by the study. The insurance applies to the damage that becomes apparent during the study or within 4 years after the end of the study.

### Outcomes {12}

#### Endpoints phase Ib

In this phase, safety and toxicity will be defined by the MTD, which is determined by assessing DLTs. A DLT is defined as any unexpected adverse event or serious adverse event deemed related to the investigational combination treatment. DLT observation period ranges from week 0 to week 12 after M-PHP plus ipilimumab and nivolumab infusion.

**Fig. 3:**
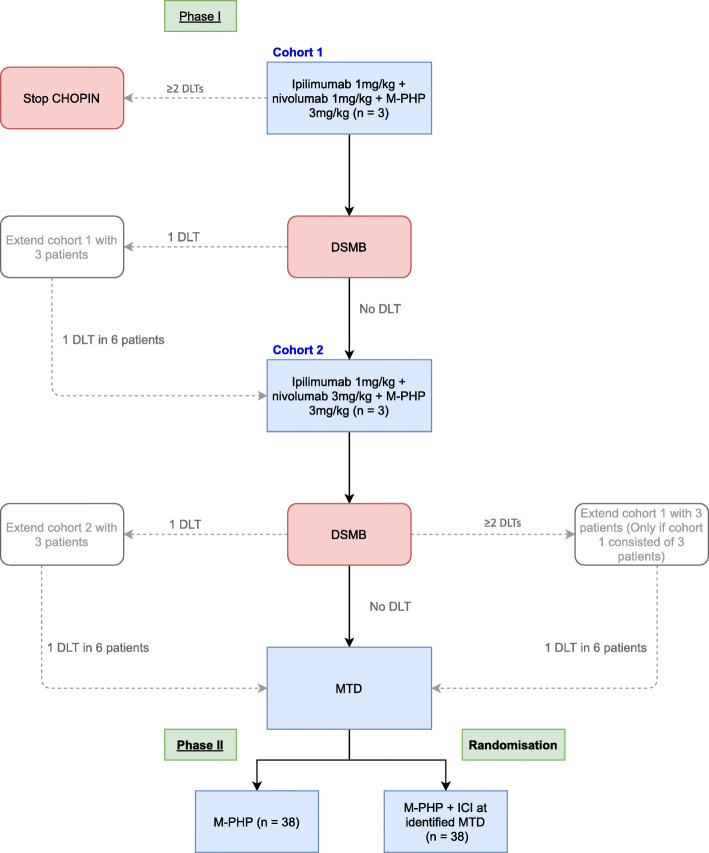
Flowchart establishment of MTD. DLT, dose limiting toxicity; MTD, maximum tolerated dose; DSMB, Data Safety Monitoring Board; ICI, immune checkpoint inhibitor (ipilimumab + nivolumab); M-PHP, percutaneous hepatic perfusion with melphalan

The flowchart for the establishment of the MTD is depicted in Fig. 3. If in the first cohort no DLT is observed in three patients, the second cohort will be opened. If two or more patients have DLTs, the study will be terminated without having identified the MTD. The procedure following possible DLTs in the first and second cohorts is depicted in Fig. 3.

#### Endpoints phase II

The primary endpoint is progression-free survival (PFS) at one year. Secondary study endpoints are best ORR according to RECIST 1.1 and irRECIST [24, 25] and OS.

PFS and OS will be assessed using Kaplan-Meier curves. Median values of OS along with 2-sided 95% CI will be calculated. OS rates at selected time points, including survival rates at years 1 and 2, together with their 95% CIs will also be estimated using Kaplan-Meier estimates. Adverse events from date of enrolment up to the last contact with patients will be presented for the full study duration and separately for events that occur on- or post-treatment.

### Participant timeline {13}

#### Screening phase

The schedule of the study assessments can be found in Table 1. Following the signing of the IC form for screening and enrolment in the study, the remainder of screening procedures and tests will be performed. During screening and before the start of treatment, blood samples will be collected and isolated peripheral blood mononuclear cell (PBMC) as well as serum/plasma samples will be cryopreserved until further analysis.

**Table 1:** Schedule of enrolment, interventions, and assessments

Week	− 4 until − 1	− 1	0	1	2	3	6	7	9	12	24	Treatment discontinuation/disease progression
History^1^	X		X			X	X		X	X	X	X
Physical examination ^2^	X		X			X	X		X	X	X	X
Viral serology ^3^	X											
Pregnancy test ^4^	X		X			X	X		X			
Hematology and blood chemistry ^5^	X		X			X	X		X	X	X	X
ECG	X											
Imaging ^6^	X						X			X	X	X
Ipilimumab/Nivolumab i.v., every 3 weeks for 4 courses^7^			X			X	X		X			
PHP^7^				X				X				
PBMC and EDTA blood^8^	X						X			X		X
Biopsy metastasis^9^	X						X					X

*ECG* electrocardiogram, *i.v.* intravenous, *Hb* hemoglobin, *Ht* hematocrit, *ANC* absolute neutrophil count, *PT* prothrombin time, *INR* international normalized ratio, *APTT* activated partial thromboplastin time, *LDH* lactate dehydrogenase, *AST* aspartate aminotransferase, *ALT* alanine aminotransferase, *GGT* gamma-glutamyl transferase, *TSH* thyroid-stimulating hormone, *fT4* free thyroxine, *CRP* C-reactive protein, *ESR* erythrocyte sedimentation rate, *RECIST 1.1.* Response Evaluation Criteria in Solid Tumors version 1.1, *DLT* dose limiting toxicity, *PBMC* peripheral blood mononuclear cell, *FFPE* formalin-fixed paraffin-embedded

^1^Histological confirmation of UM liver metastases

^2^Including the assessment of patients’ height, weight, performance status, and vital signs

^3^HIV antibody titer, HbsAg determination, Anti-HCV, anti-CMV antibody titer

^4^For female patients of child bearing age only

^5^Hematology: Hb, platelet count, absolute neutrophil count, white blood cell diff, hematocrit, PT/INR, APTT. Chemistry: LDH, phosphorus, sodium, potassium, magnesium, chloride, calcium, creatinine, albumin, total protein, AST, ALT, bilirubin (indirect + direct), GGT, alkaline phosphatase, glucose, amylase, lipase, TSH, fT4, cortisol, CRP, ESR

^6^CT of the chest and abdomen, and MRI of the liver (if liver metastases are not measurable according to RECIST 1.1 on CT scan) to assess the number and size of metastases. Lesions must be defined according to RECIST version 1.1. Ideally, initial imaging is performed as closely as possible to the first ipilimumab/nivolumab infusion, but never more than 4 weeks apart. Thereafter, patients should be evaluated with CT/MRI scans every 3 months in year 1, every 4 months in years 2 and 3, and every 6 months in years 4 and 5

^7^We will first start with four courses of ipilimumab 1 mg/kg and nivolumab 1 mg/kg and two M-PHP-procedures. In case of a safe application according to the criteria described in the cohort/DLT-section, we will continue with 4 courses of ipilimumab 1 mg/kg and nivolumab 3 mg/kg and two M-PHP-procedures.

^8^PBMC’s and EDTA blood (for isolation of plasma and thrombocytes) will be taken twice before the start of treatment. Furthermore, PBMC’s will be collected in week 6, and week 12, and in case of tumor relapse/disease progression

^9^Liver biopsies will be performed prior to treatment, in week 6 and in case of tumor relapse/disease progression (optional), 3× 14g: 2× frozen, 1× FFPE for additional molecular biological and immunological tests

#### During treatment

Targeted physical examination and standard blood tests will be regularly performed (hematology and chemistry). A second liver tumor biopsy is taken at week 6. Computed tomography (CT) scans of chest and abdomen are performed in weeks 6, 12, and 24. The treating physician will record in their standardized clinical notes any observed AEs during the course of treatment and follow-up.

#### Follow-up procedure

Follow-up will consist of a three-monthly physical examination, blood tests and CT scans of the chest and abdomen during the first year (and MRI of the liver if liver metastases are not measurable according to RECIST 1.1 on CT-scan). In the second and third years, the same evaluations are performed approximately every four months and then every 6 months in the following years. In case of disease progression, PBMCs and tumor biopsies will be collected.

### Sample size {14}

Phase Ib will consist of a minimum of 3 and maximum of 12 patients, according to the dose-escalation schedule and flowchart previously described (Figs. 2 and 3). In the randomized phase II part of the study, we aim to demonstrate the superiority of combined M-PHP plus ipilimumab and nivolumab over M-PHP only based on the assumption that PFS at 1 year will increase from 20% in the M-PHP arm to 50% in the combination arm. Using a one-sided α of 5% and 80% power (*β*), this requires 38 patients in each arm (*α*=0.05, *β*=0.20, P0=20%, P1=50%), resulting in a total of 76 patients. In total, 79–88 patients will be treated with the optimal dose in phases Ib and II combined.

### Recruitment {15}

The LUMC is the main referral center in the Netherlands for UM patients and is currently the only center in the Netherlands that performs the M-PHP procedures. Patients will be recruited in oncology centers in the Netherlands and referred to the LUMC for treatment. The trial is currently recruiting patients. The expected recruitment rate is 20–25 patients per year, with a treatment duration of 24 weeks per patient. Based on this the estimated study completion date is in December 2024.

## Assignment of interventions: allocation

### Sequence generation {16a}

This is an open-label trial. In phase II patients will be randomized on a 1:1 basis to receive M-PHP with ICI or M-PHP only. Randomization is performed by means of a computerized randomization schedule via Castor Electronic Data Capture.

### Concealment mechanism {16b}

Allocation is concealed by assigning the treatment group to the patient until after recruitment and signing of IC. Considering the nature of the two treatment arms it is not possible to blind either the patients or the physicians/research team.

### Implementation {16c}

Sequence generation will be performed by means of the computerized program Castor Electronic Data Capture by a person that does not have a therapeutic relationship with the trial participants.

## Assignment of interventions: Blinding

### Who will be blinded {17a}

This is an open-label trial, blinding is not applicable.

### Procedure for unblinding if needed {17b}

This trial is unblinded, therefore no unblinding procedure is needed.

## Data collection and management

### Plans for assessment and collection of outcomes {18a}

The treating physician will give the IC form to patients. Baseline information is collected via the oncology and radiology departments, by means of laboratory testing for blood samples, imaging, and biopsies. Data is inserted in the electronic database by the data manager.

### Plans to promote participant retention and complete follow-up {18b}

Patients are adequately informed prior to attending the trial and are followed up according to a detailed follow-up plan.

### Data management {19}

All data that are relevant for the study will be collected on case report forms (CRFs). The CRFs must be reviewed, signed, and dated by the Principal Investigator (PI) or sub-investigator. The CRFs will be kept at the LUMC. Entered data will be cleaned using consistency checks according to a Data Validation Plan. After complete cleaning of the data, a quality control check will be performed. When the result of this check is satisfactory, the database will be locked. All data management procedures will be documented in detail in a study-specific Data Management Plan.

### Confidentiality {27}

The handling of personal data complies with the Dutch General Regulation Data Protection. Uncoded data will be available to the study monitors and the Dutch Health and Youth Care Inspectorate for inspection purposes. Data will be stored for a maximum of 15 years. Patient material (tumor and blood material) will be stored for a maximum of 15 years. The involved research team can work with the material. The handling of personal data complies with the Dutch General Regulation Data Protection. After that period, it will be destroyed under responsibility of the PI. At the request of the patient, the material will be destroyed earlier.

### Plans for collection, laboratory evaluation, and storage of biological specimens for genetic or molecular analysis in this trial/future use {33}

Patient material (tumor and blood material) will be stored for a maximum of 15 years to be used for additional (immunological) research in line with the current research question. New technological advancements and new insights may result in new questions related to the improvement of treatment for melanoma patients, for which the isolated patient materials are uniquely suited as test material.

## Statistical methods

### Statistical methods for primary and secondary outcomes {20a}

Baseline characteristics will be described for all patients in phases Ib and II. Continuous variables will be compared with a t-test and categorical variables with the Chi-square test. To study the association between risk factors and toxicity a logistic regression model will be estimated. Analyses will be performed after treatment in the cohorts in the phase Ib part and after treatment of all included patients at the end of phase II.

OS is defined as the time between the start of treatment and the date of death due to any cause. A patient who has not died will be censored at the last known date alive. Patients will be followed up while on the study drug and every 4 weeks via in-person or phone contact after discontinuation of the study. PFS and OS will be assessed using Kaplan-Meier curves. Median values of OS along with 2-sided 95% CI will be calculated. OS rates at selected time points, including survival rates at years 1 and 2, together with their 95% CIs will also be estimated using Kaplan-Meier estimates. Adverse events from the date of enrolment up to the last contact with patients will be presented for the full study duration and separately for events that occur on- or post-treatment. These AEs will include the safety endpoints, high-grade, treatment-related select AEs and their characteristics, and all other AEs. In particular, safety data will be summarized and listed for all treated. All on-study AEs, drug-related AEs, serious adverse events (SAEs), and drug-related SAEs will be tabulated using the worst grade per CTCAE version 4.03 criteria.

### Interim analyses {21b}

A conditional power analysis will be performed mid-way. In total 79-82 patients will be treated with the optimal dose in phases I and II combined. Three months after the start of treatment of the 40th patient all data on PFS of the included patients will be analyzed. The conditional power will be used as the basis for potential early termination for futility when there is little evidence of a beneficial effect. The stopping rule will be set at a conditional power of 10%. However, the decision on early termination of the trial will be based on the opinion of the Data Safety Monitoring Board (DSMB) after consulting the principle investigator.

### Methods for additional analyses (e.g., subgroup analyses) {20b}

OS summary statistics by subgroups will also be reported.

### Methods in analysis to handle protocol non-adherence and any statistical methods to handle missing data {20c}

Study participation status including completion and discontinuation of treatment will be reported. Reasons for discontinuation will be summarized. Inevitable missing data, such as withdrawals or loss to follow-up data will be analyzed according to the intention-to-treat principle.

### Plans to give access to the full protocol, participant level-data and statistical code {31c}

The protocol information is accessible via the trial registry website.

## Oversight and monitoring

### Composition of the coordinating center and trial steering committee {5d}

This is a single-center study. The PI and research team are in charge of patient recruitment, data collection, CRF completion, and patient follow-up. Additionally, the DSMB will guard the safety of the procedures and the monitoring committee will frequently monitor the study.

### Composition of the data monitoring committee, its role, and reporting structure {21a}

The monitoring committee of the LUMC will be in charge of frequent monitor visits to guard the completeness of acquired study data and quality assurance of the trial. Monitor visits are conducted by a dedicated research monitor. This study is considered a high-risk study.

A DSMB is installed that will meet after treatment of every cohort in the phase Ib part and after treatment of 3patients who have been randomized to the combination treatment of M-PHP with ipilimumab and nivolumab, to evaluate the safety and toxicity of treatment. The DSMB consists of a medical oncologist, an interventional radiologist, and a statistician. All members have experience with M-PHP and/or immunotherapy and/or clinical trials, but are not involved in the described clinical protocol. This is required for the objective evaluation of the safety and toxicity of treatment. The advice(s) of the DSMB will be sent to the sponsor of the study. Should the sponsor decide not to fully implement the DSMB advice, they will send the advice to the reviewing MREC, including a note to substantiate why (part of) the advice of the DSMB will not be followed.

### Adverse event reporting and harms {22}

The investigator will report all SAEs to BMS and Delcath within 24 hours of knowledge, including pregnancy, overdose, secondary cancer, drug-induced liver injury, and other severe laboratory test abnormalities. The investigator will report the SAEs through the web portal ToetsingOnline to the MREC, within 7 days of first knowledge for SAEs that result in death or are life-threatening followed by a period of a maximum of 8 days to complete the initial preliminary report. All other SAEs will be reported within a period of maximum 15 days after the sponsor has first knowledge of the serious adverse events.

### Frequency and plans for auditing trial conduct {23}

At the start of the trial, an initiation visit is planned with the monitoring committee. Source data verification of the CRFs and check of the Investigator Study File documents will be performed by the clinical research monitor, according to the procedures described in the Monitor Plan and in the heading “ Composition of the data monitoring committee, its role, and reporting structure”.

### Plans for communicating important protocol amendments to relevant parties (e.g., trial participants, ethical committees) {25}

All substantial amendments will be notified to the MREC and to the competent authority.

Non-substantial amendments will not be notified to the MREC and competent authority, but will be recorded and filed by the investigator.

### Dissemination plans {31a}

The results from this trial will be submitted to a peer-reviewed journal for publication. Data will be published anonymously.

## Discussion

This is the first trial to evaluate the effect of M-PHP combined with ipilimumab and nivolumab in UM patients. The MTD of the combination treatment will be defined in phase Ib. Subsequently, the efficacy of the combination of M-PHP and ICI will be compared to M-PHP only in a randomized phase II trial.

ICI revolutionized the treatment of patients with CM in the past years. Treatment with ICI relies on activation of antigen-specific T cells by inhibiting their normal immunoregulatory mechanisms [12, 26]. Updated survival data from the Checkmate 067 trial after a minimum follow-up of 5 years showed the supremacy of nivolumab either alone or in combination with ipilimumab over ipilimumab monotherapy in CM. The five-year OS was 52% in patients that were treated with ipilimumab plus nivolumab, compared to 44% and 26% in the nivolumab only and ipilimumab only groups respectively [27]. Several studies evaluated the effect of anti-CTLA-4 and anti-PD-1 monoclonal antibodies in CM patients [2, 9–11, 26], but results of ICI in patients with UM have been disappointing. Possible reasons for the lower response rates of ICI in metastatic UM may include the low mutational burden and therefore the limited expression of neoantigens recognizable by cytotoxic T cells. One explanation for this difference is the lack of ultraviolet-radiation damage in UM, when compared to CM [12]. Additionally, only about 10% of UM primary tumors [28] and 5% of the UM cells in metastatic disease express PD-L1 [29]. Another factor is that UM arises in an immune-privileged environment, that possesses inhibitory properties against both the innate and the adaptive immune system [30]. Finally, it is postulated that the liver functions as an immune-modulating organ, possibly enhancing tolerance to tumor antigens [2, 31]. To improve the efficacy of ICI, broadening of the T cell repertoire is necessary. Combining ipilimumab and nivolumab might overcome the need for a high mutational load [32]. Recent studies confirm that combination treatment shows higher response rates than single-agent treatment with either PD-1 or CTLA-4 inhibitors alone in UM patients. A recent large retrospective trial including both pre-treated and treatment-naïve patients with metastatic uveal melanoma showed an ORR of 11.6% and disease control rate of 36.0% (median OS of 15 months) [14]. These results were comparable with those of a phase II trial, with an ORR of 18% and clinical benefit rate of 37% (median OS 19.1 months) [15]. In the GEM-1402 study, a phase II trial conducted on treatment-naïve metastatic UM patients, a similar objective response rate of 11.5% was found, with a higher disease control rate of 63.5% and a median OS of 12.7 months. Interestingly, in this trial patients with extrahepatic disease showed a longer survival than patients with metastases located in the liver (23.5 months compared to 9.2 months). Even though this difference was not statistically significant, it suggests that patients with extrahepatic disease could better benefit from combination treatment with ipilimumab and nivolumab compared to those with hepatic metastases [16].

As the liver is the dominant predilection site for UM metastases and survival correlates with disease control in the liver, a variety of liver-directed therapies have been studied. While published literature varies considerably in terms of patient selection, disease extent, design, and outcome measurements, it suggests that locoregional liver therapies offer survival benefit in selected patients with hepatic metastases [33–36].

Locoregional therapies may induce immune responses and trials are trying to exploit the potential synergistic effect of combined local liver therapy with ICI in melanoma patients. However, the number of studies investigating such combination therapy in UM is limited up until now*.* A retrospective study evaluated a combination treatment of transarterial radioembolizaton (TARE) with CTLA-4 or PD-1 antibody therapy showed promising results. The study demonstrated a median hepatic PFS of 15.0 months and OS of 17.0 months after the start of TARE, with a local disease control rate of 63.6%. However, no standard treatment regimen was applied and the study sample size was small [37]*.* A recent prospective, phase Ib/II trial showed that combining radio-frequency ablation (RFA) with ipilimumab 3 mg/kg was well tolerated by patients. This resulted nonetheless in very limited clinical activity, measured by a low disease control rate of 7%, a 6-month PFS of 7%, and a 1- and 2-year OS of 51% and 7%, respectively [38]. A phase II trial combining ipilimumab and nivolumab with immuno-embolization in metastatic UM patients is on-going (recruiting, trial number NCT03472586).

In the present trial, we are combining M-PHP with ICI. M-PHP has become the standard of care in our institution, showing safe and favorable outcomes in patients with hepatic metastases of UM [39, 40]. The principle of M-PHP is to isolate the venous return of the liver from the systemic circulation. The blood returning from the liver is purified from melphalan via an extracorporeal circuit and returned to the patient. This allows perfusion of the liver with a very high dose of melphalan, which would be toxic and would lead to severe complications when administered systemically. The superiority of M-PHP over best alternative care has been demonstrated in a randomized controlled multicenter phase III trial for patients with liver metastases from CM and UM [18]. In this trial, the hepatic PFS and OS in the M-PHP group were 7.0 and 5.4 months respectively, compared to 1.6 and 1.6 months, respectively, in the best alternative care group. In a prospective, phase II trial including 35 patients with UM metastases confined to the liver, we found a 72% objective response rate after M-PHP and a median OS of 19.1 months [19]. A recent retrospective study on M-PHP in 51 patients with metastatic UM demonstrated a slightly lower median OS of 15.3 months. However, this study also included patients with limited extrahepatic metastases, which could explain the difference [20]. To date little is known on immunomodulation by M-PHP. The available evidence stems mainly from studies on Isolated Limb Perfusion (ILP) [41] in metastatic CM patients and Isolated Hepatic Perfusion (IHP) [42] in metastatic UM patients, which are two procedures with a similar principle as M-PHP. In these studies, it was concluded that the efficacy of ILP and IHP rely, in part, on T cell activation following the procedure. The authors hypothesize that immunomodulation by IHP may help to overcome the low immunogenicity of UM and turn “cold tumors” into “hot tumors” targetable by immunotherapy. Exploratory end-points are included in our study to analyze immunological parameters and changes in tumor immune-infiltrates, PBMC, and serum.

## Conclusion

Patients with metastasized UM have a poor prognosis and no standard treatment options. When it comes to metastatic disease to the liver, M-PHP is an effective treatment. However, the majority of patients will eventually develop the extrahepatic disease. In order to improve the clinical outcome for metastatic UM patients, we have initiated this phase Ib/randomized phase II clinical trial combining M-PHP with the ICIs ipilimumab (anti-CTLA4 monoclonal antibody) and nivolumab (anti-PD-1 monoclonal antibody). This combination might induce a synergistic effect, leading to a better and sustained treatment response both in and outside the liver.

## Trial status

This trial was registered in the U.S. National Library of Medicine with identifier NCT04283890 and is currently recruiting participants. Enrolment for phase Ib started in December 2019. The current study protocol is version 6. All significant protocol amendments will be submitted to the MREC and will be submitted to the trial registration website www.ClinicalTrials.gov. Estimated study completion is in December 2024.

## Abbreviations

AE: Adverse event
AR: Adverse reaction
CA: Competent Authority
CCMO: Central Committee on Research Involving Human Subjects
CM: Cutaneous melanoma
CR: Complete response
CT: Computed tomography
CTCAE v 4.03: Common Terminology Criteria for Adverse Events version 4.03
DCR: Disease control rate
DLT: Dose limiting toxicity
DSMB: Data Safety Monitoring Board
EDTA: Ethylenediamine tetraacetic acid
GCP: Good Clinical Practice
IC: Informed consent
ICH: International Council for Harmonisation of Technical Requirements for Pharmaceuticals for Human Use
ICI: Immune checkpoint inhibitor
irRECIST: Immune-related Response Evaluation Criteria in Solid Tumors
M-PHP: Percutaneous Hepatic Perfusion with Melphalan
MREC: Medical Research Ethics Committee
MRI: Magnetic resonance imaging
MTD: Maximum tolerated dose
UM: Uveal melanoma
ORR: Overall response rate
OS: Overall survival
PBMC: Peripheral blood mononuclear cell
PD: Progressive disease
PFS: Progression-free survival
PI: Principal Investigator
PR: Partial response
RECIST 1.1: Response Evaluation Criteria in Solid Tumors version 1.1
RFA: Radio-frequency ablation
RPTD: Recommended phase II dose
SAE: Serious adverse event
SD: Stable disease
SIRT: Selective internal radiation therapy
TARE: Trans-arterial radio-embolization
WHO: World Health Organization

## References

[1] Singh AD, Turell ME, Topham AK (2011). Uveal melanoma: trends in incidence, treatment, and survival. Ophthalmology.

[2] Wessely A, Steeb T, Erdmann M, Heinzerling L, Vera J, Schlaak M (2020). The Role of Immune Checkpoint Blockade in Uveal Melanoma. Int J Mol Sci.

[3] Heppt MV, Steeb T, Schlager JG, Rosumeck S, Dressler C, Ruzicka T, Nast A, Berking C (2017). Immune checkpoint blockade for unresectable or metastatic uveal melanoma: A systematic review. Cancer Treat Rev.

[4] Xu LT, Funchain PF, Bena JF, Li M, Tarhini A, Berber E, Singh AD (2019). Uveal Melanoma Metastatic to the Liver: Treatment Trends and Outcomes. Ocul Oncol Pathol.

[5] Diener-West M, Reynolds SM, Agugliaro DJ, Caldwell R, Cumming K, Earle JD, Hawkins BS, Hayman JA, Jaiyesimi I, Jampol LM, Kirkwood JM, Koh WJ, Robertson DM, Shaw JM, Straatsma BR, Thoma J, Collaborative Ocular Melanoma Study Group (2005). Development of metastatic disease after enrollment in the COMS trials for treatment of choroidal melanoma: Collaborative Ocular Melanoma Study Group Report No. 26. Arch Ophthalmol.

[6] Hodi FS, O'Day SJ, McDermott DF, Weber RW, Sosman JA, Haanen JB (2010). Improved survival with ipilimumab in patients with metastatic melanoma. N Engl J Med.

[7] Larkin J, Chiarion-Sileni V, Gonzalez R, Grob JJ, Cowey CL, Lao CD, Schadendorf D, Dummer R, Smylie M, Rutkowski P, Ferrucci PF, Hill A, Wagstaff J, Carlino MS, Haanen JB, Maio M, Marquez-Rodas I, McArthur GA, Ascierto PA, Long GV, Callahan MK, Postow MA, Grossmann K, Sznol M, Dreno B, Bastholt L, Yang A, Rollin LM, Horak C, Hodi FS, Wolchok JD (2015). Combined Nivolumab and Ipilimumab or Monotherapy in Untreated Melanoma. N Engl J Med.

[8] Robert C, Schachter J, Long GV, Arance A, Grob JJ, Mortier L, Daud A, Carlino MS, McNeil C, Lotem M, Larkin J, Lorigan P, Neyns B, Blank CU, Hamid O, Mateus C, Shapira-Frommer R, Kosh M, Zhou H, Ibrahim N, Ebbinghaus S, Ribas A (2015). Pembrolizumab versus Ipilimumab in Advanced Melanoma. N Engl J Med.

[9] van der Kooij MK, Joosse A, Speetjens FM, Hospers GA, Bisschop C, de Groot JW (2017). Anti-PD1 treatment in metastatic uveal melanoma in the Netherlands. Acta Oncol.

[10] Algazi AP, Tsai KK, Shoushtari AN, Munhoz RR, Eroglu Z, Piulats JM, Ott PA, Johnson DB, Hwang J, Daud AI, Sosman JA, Carvajal RD, Chmielowski B, Postow MA, Weber JS, Sullivan RJ (2016). Clinical outcomes in metastatic uveal melanoma treated with PD-1 and PD-L1 antibodies. Cancer.

[11] Kelderman S, van der Kooij MK, van den Eertwegh AJ, Soetekouw PM, Jansen RL, van den Brom RR (2013). Ipilimumab in pretreated metastastic uveal melanoma patients. Results of the Dutch Working group on Immunotherapy of Oncology (WIN-O). Acta Oncol.

[12] van der Kooij MK, Speetjens FM, van der Burg SH, Kapiteijn E (2019). Uveal Versus Cutaneous Melanoma; Same Origin, Very Distinct Tumor Types. Cancers (Basel).

[13] Heppt MV, Heinzerling L, Kahler KC, Forschner A, Kirchberger MC, Loquai C (2017). Prognostic factors and outcomes in metastatic uveal melanoma treated with programmed cell death-1 or combined PD-1/cytotoxic T-lymphocyte antigen-4 inhibition. Eur J Cancer.

[14] Najjar YG, Navrazhina K, Ding F, Bhatia R, Tsai K, Abbate K (2020). Ipilimumab plus nivolumab for patients with metastatic uveal melanoma: a multicenter, retrospective study. J Immunother Cancer.

[15] Pelster MS, Gruschkus SK, Bassett R, Gombos DS, Shephard M, Posada L, Glover MS, Simien R, Diab A, Hwu P, Carter BW, Patel SP (2021). Nivolumab and Ipilimumab in Metastatic Uveal Melanoma: Results From a Single-Arm Phase II Study. J Clin Oncol.

[16] Piulats JM, Espinosa E, de la Cruz ML, Varela M, Alonso Carrion L, Martin-Algarra S (2021). Nivolumab Plus Ipilimumab for Treatment-Naive Metastatic Uveal Melanoma: An Open-Label, Multicenter, Phase II Trial by the Spanish Multidisciplinary Melanoma Group (GEM-1402). J Clin Oncol.

[17] Eschelman DJ, Gonsalves CF, Sato T (2013). Transhepatic therapies for metastatic uveal melanoma. Semin Intervent Radiol.

[18] Hughes MS, Zager J, Faries M, Alexander HR, Royal RE, Wood B, Choi J, McCluskey K, Whitman E, Agarwala S, Siskin G, Nutting C, Toomey MA, Webb C, Beresnev T, Pingpank JF (2015). Results of a Randomized Controlled Multicenter Phase III Trial of Percutaneous Hepatic Perfusion Compared with Best Available Care for Patients with Melanoma Liver Metastases. Ann Surg Oncol.

[19] Meijer TS, Burgmans MC, de Leede EM, de Geus-Oei LF, Boekestijn B, Handgraaf HJM (2020). Percutaneous Hepatic Perfusion with Melphalan in Patients with Unresectable Ocular Melanoma Metastases Confined to the Liver: A Prospective Phase II Study. Ann Surg Oncol.

[20] Karydis I, Gangi A, Wheater MJ, Choi J, Wilson I, Thomas K, Pearce N, Takhar A, Gupta S, Hardman D, Sileno S, Stedman B, Zager JS, Ottensmeier C (2018). Percutaneous hepatic perfusion with melphalan in uveal melanoma: A safe and effective treatment modality in an orphan disease. J Surg Oncol.

[21] Pingpank JF, Libutti SK, Chang R, Wood BJ, Neeman Z, Kam AW, Figg WD, Zhai S, Beresneva T, Seidel GD, Alexander HR (2005). Phase I study of hepatic arterial melphalan infusion and hepatic venous hemofiltration using percutaneously placed catheters in patients with unresectable hepatic malignancies. J Clin Oncol.

[22] de Leede EM, Burgmans MC, Meijer TS, Martini CH, Tijl FGJ, Vuyk J, van Erkel AR, van der Velde CJH, Kapiteijn E, Vahrmeijer AL (2017). Prospective Clinical and Pharmacological Evaluation of the Delcath System's Second-Generation (GEN2) Hemofiltration System in Patients Undergoing Percutaneous Hepatic Perfusion with Melphalan. Cardiovasc Intervent Radiol.

[23] Kirstein MM, Marquardt S, Jedicke N, Marhenke S, Koppert W, Manns MP, Wacker F, Vogel A (2017). Safety and efficacy of chemosaturation in patients with primary and secondary liver tumors. J Cancer Res Clin Oncol.

[24] Nishino M, Giobbie-Hurder A, Gargano M, Suda M, Ramaiya NH, Hodi FS (2013). Developing a common language for tumor response to immunotherapy: immune-related response criteria using unidimensional measurements. Clin Cancer Res.

[25] Seymour L, Bogaerts J, Perrone A, Ford R, Schwartz LH, Mandrekar S, Lin NU, Litière S, Dancey J, Chen A, Hodi FS, Therasse P, Hoekstra OS, Shankar LK, Wolchok JD, Ballinger M, Caramella C, de Vries EGE, RECIST working group (2017). iRECIST: guidelines for response criteria for use in trials testing immunotherapeutics. Lancet Oncol.

[26] Singh BP, Salama AK (2016). Updates in Therapy for Advanced Melanoma. Cancers (Basel).

[27] Larkin J, Chiarion-Sileni V, Gonzalez R, Grob JJ, Rutkowski P, Lao CD, Cowey CL, Schadendorf D, Wagstaff J, Dummer R, Ferrucci PF, Smylie M, Hogg D, Hill A, Márquez-Rodas I, Haanen J, Guidoboni M, Maio M, Schöffski P, Carlino MS, Lebbé C, McArthur G, Ascierto PA, Daniels GA, Long GV, Bastholt L, Rizzo JI, Balogh A, Moshyk A, Hodi FS, Wolchok JD (2019). Five-Year Survival with Combined Nivolumab and Ipilimumab in Advanced Melanoma. N Engl J Med.

[28] Kaunitz GJ, Cottrell TR, Lilo M, Muthappan V, Esandrio J, Berry S, Xu H, Ogurtsova A, Anders RA, Fischer AH, Kraft S, Gerstenblith MR, Thompson CL, Honda K, Cuda JD, Eberhart CG, Handa JT, Lipson EJ, Taube JM (2017). Melanoma subtypes demonstrate distinct PD-L1 expression profiles. Lab Invest.

[29] Javed A, Arguello D, Johnston C, Gatalica Z, Terai M, Weight RM, et al. PD-L1 expression in tumor metastasis is different between uveal melanoma and cutaneous melanoma. 2017. 10.2217/imt-2017-0066. 10.2217/imt-2017-0066 29185395

[30] Taylor AW (2016). Ocular Immune Privilege and Transplantation. Front Immunol.

[31] Doherty DG (2016). Immunity, tolerance and autoimmunity in the liver: A comprehensive review. J Autoimmun.

[32] Rozeman EA, Fanchi L, van Akkooi ACJ, Kvistborg P, Thienen JV, Stegenga B, et al. (Neo-)adjuvant ipilimumab + nivolumab (IPI+NIVO) in palpable stage 3 melanoma – updated relapse free survival (RFS) data from the OpACIN trial and first biomarker analyses. Ann Oncol. 2017;28. 10.1093/annonc/mdx377.008.

[33] Burgmans MC, de Leede EM, Martini CH, Kapiteijn E, Vahrmeijer AL, van Erkel AR (2016). Percutaneous Isolated Hepatic Perfusion for the Treatment of Unresectable Liver Malignancies. Cardiovasc Intervent Radiol.

[34] de Leede EM, Burgmans MC, Martini CH, Tijl FG, van Erkel AR, Vuyk J, et al. Percutaneous Hepatic Perfusion (PHP) with Melphalan as a Treatment for Unresectable Metastases Confined to the Liver. J Vis Exp. 2016;113. 10.3791/53795 PMC5091707 27501370

[35] Rowcroft A, Loveday BPT, Thomson BNJ, Banting S, Knowles B (2020). Systematic review of liver directed therapy for uveal melanoma hepatic metastases. HPB (Oxford).

[36] Gonsalves CF, Adamo RD, Eschelman DJ (2020). Locoregional Therapies for the Treatment of Uveal Melanoma Hepatic Metastases. Semin Intervent Radiol.

[37] Zheng J, Irani Z, Lawrence D, Flaherty K, Arellano RS (2018). Combined Effects of Yttrium-90 Transarterial Radioembolization around Immunotherapy for Hepatic Metastases from Uveal Melanoma: A Preliminary Retrospective Case Series. J Vasc Interv Radiol.

[38] Rozeman EA, Prevoo W, Meier MAJ, Sikorska K, Van TM, van de Wiel BA (2020). Phase Ib/II trial testing combined radiofrequency ablation and ipilimumab in uveal melanoma (SECIRA-UM). Melanoma Res.

[39] Meijer TS, Geus-Oei LF, Martini CH, Tijl FGJ, Sitsen ME, Erkel ARV (2019). Embolization of variant hepatic arteries in patients undergoing percutaneous hepatic perfusion for unresectable liver metastases from ocular melanoma. Diagn Interv Radiol.

[40] Meijer TS, Burgmans MC, Fiocco M, de Geus-Oei LF, Kapiteijn E, de Leede EM, Martini CH, van der Meer RW, Tijl FGJ, Vahrmeijer AL (2019). Safety of Percutaneous Hepatic Perfusion with Melphalan in Patients with Unresectable Liver Metastases from Ocular Melanoma Using the Delcath Systems' Second-Generation Hemofiltration System: A Prospective Non-randomized Phase II Trial. Cardiovasc Intervent Radiol.

[41] Johansson J, Kiffin R, Andersson A, Lindnér P, Naredi PL, Olofsson Bagge R, et al. Isolated Limb Perfusion With Melphalan Triggers Immune Activation in Melanoma Patients. Front Oncol. 2018;8. 10.3389/fonc.2018.00570. 10.3389/fonc.2018.00570 PMC6286961 30560089

[42] Johansson J, Siarov J, Kiffin R, Molne J, Mattsson J, Naredi P (2020). Presence of tumor-infiltrating CD8(+) T cells and macrophages correlates to longer overall survival in patients undergoing isolated hepatic perfusion for uveal melanoma liver metastasis. Oncoimmunology.

